# Simultaneous triple primary malignancies, including bladder cancer, lymphoma, and lung cancer, in an elderly male: A case report

**DOI:** 10.1515/biol-2022-0500

**Published:** 2022-09-26

**Authors:** Risheng Huang, Zhijia Li, Shanshan Weng, Shenghao Wu

**Affiliations:** Department of Thoracic Surgery, The Dingli Clinical College of Wenzhou Medical University (The Second Affiliated Hospital of Shanghai University, Wenzhou Central Hospital), Wenzhou City, Zhejiang Province, China; Department of Urology Surgery, The Dingli Clinical College of Wenzhou Medical University (The Second Affiliated Hospital of Shanghai University, Wenzhou Central Hospital), Wenzhou City, Zhejiang Province, China; Department of Hematology, The Dingli Clinical College of Wenzhou Medical University (The Second Affiliated Hospital of Shanghai University Wenzhou Central Hospital), Wenzhou City, Zhejiang Province, China

**Keywords:** multiple malignancies, bladder cancer, lymphoma, lung cancer

## Abstract

Multiple primary malignancies (MPMs) are defined as the coexistence of at least two unrelated primary malignancies in a single patient, with the tumors differing in their histology. MPMs in the same patient, when present within 6 months of the primary tumor diagnosis, are considered a synchronous occurrence. In this case report, we describe a 61-year-old man who presented with three distinct tumors concurrently in 2021: noninvasive urothelial carcinoma of the bladder, diffuse large B-cell lymphoma, and squamous cell carcinoma of the lung. We discuss the process of therapy and briefly review the literature. MPMs are increasing in incidence, requiring an interdisciplinary approach to diagnosis and treatment.

## 1 Introduction

Multiple primary malignancies (MPMs) are characterized by the development of two or more malignancies occurring independently in the same or different organs, excluding metastatic sites [1]. Most MPMs are reported in case reports, and clinical trials for MPMs are few and far between [2]. MPMs have been reported by single institutions and in certain countries’ registries, but their development is more complex and likely multifactorial. Prior to cancer treatment, smoking, dietary habits, and genetic mutations have been identified as risk factors for MPMs [3]. Advances in early tumor detection and better treatment have improved cancer survivorship, contributing to the increasing incidence of additional cancers [4,5]. Here, we describe a case of a 61-year-old man who was diagnosed with bladder cancer, diffuse large B-cell lymphoma (DLBCL), and lung cancer. To our knowledge, this constellation of tumors has never been reported in the literature.

## 2 Case report

**Figure 1:**
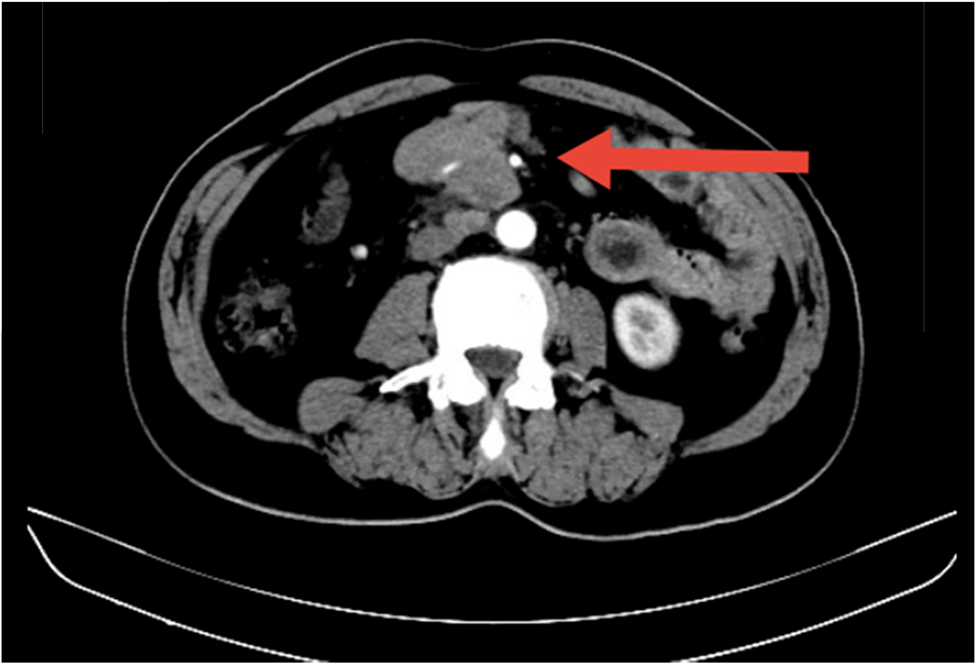
CT image revealed that the intestinal wall of some small intestine in the lower abdomen was significantly thickened and multiple lymph nodes were found in the retroperitoneal space (arrowhead).

**Figure 2:**
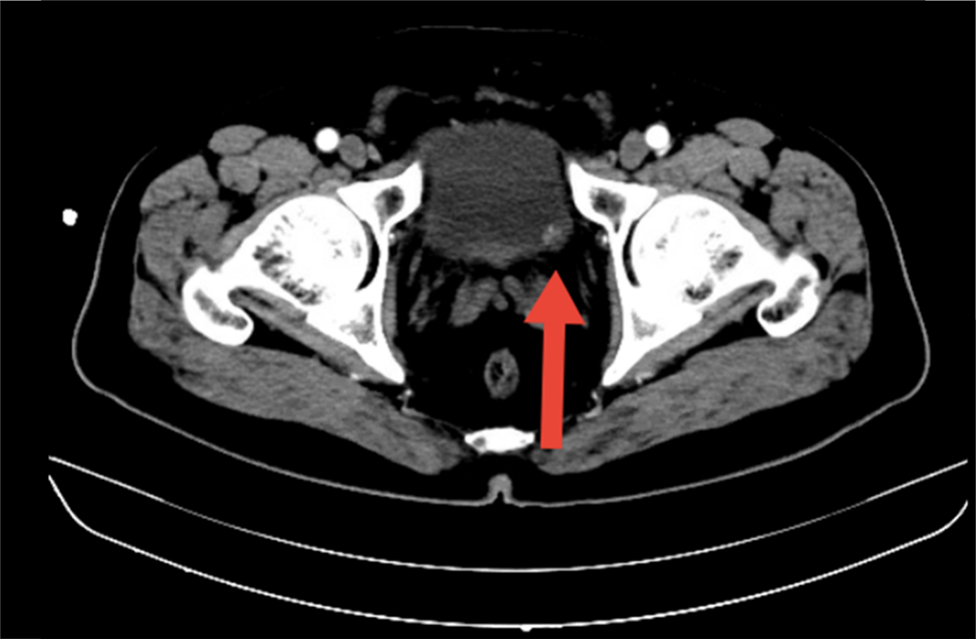
CT image revealed a nodule in the left wall of the bladder (0.8 cm × 0.8 cm).

A 61-year-old man, who was not an alcohol drinker or a tobacco user, was referred to our hospital with dull pain in his lower abdomen, which was paroxysmal and aggravated, accompanied by mild fever and loss of appetite for 3 days. He had no other cancer history except that his mother died of gastric adenocarcinoma at the age of 77 years. There were elevated white blood cell counts and elevated C-reactive protein in the laboratory data, and the serum tumor marker levels were within the normal range. Given the persistency of the symptoms, a computed tomography (CT) scan was performed, which showed that the intestinal wall of some small intestines in the lower abdomen was significantly thickened, multiple lymph nodes were found in the retroperitoneal space, and some of them were fused into clusters. The possibility of lymphoma was considered (Figure 1). CT imaging also revealed a nodule (0.8 cm × 0.8 cm) in the left wall of the bladder (Figure 2).

**Figure 3:**
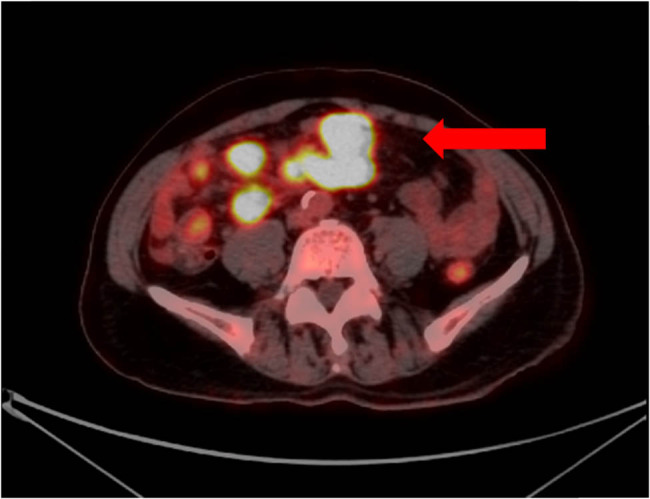
18F-FDG PET/CT image revealed that the intestinal wall of some small intestine in the lower abdomen was significantly thickened segmentally, multiple lymph nodes were found in the retroperitoneal space, some of them were fused into clusters, and the glucose metabolism was abnormally increased (arrowhead).

**Figure 4:**
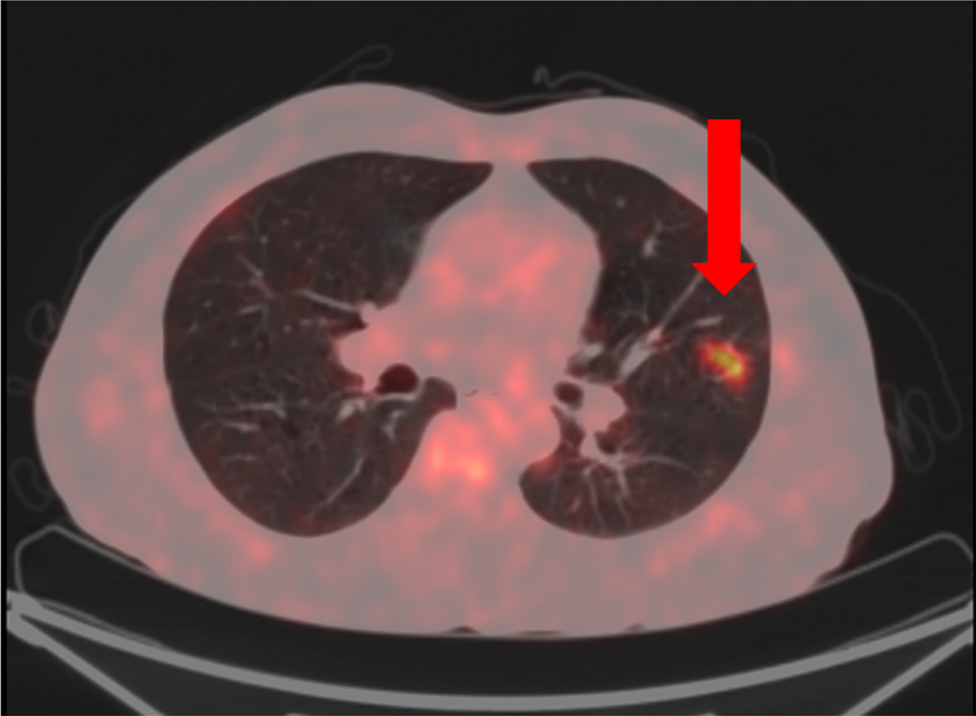
18F-FDG PET/CT image revealed a nodule (1.8 cm × 0.8 cm) in the lingula segment of the left upper lobe (arrowhead).

18F-fluorodeoxyglucose (18F-FDG) Positron emission tomography/computed tomography (PET/CT) confirmed the presence of multiple abdominal lymph nodes (Figure 3) and revealed avid uptake in the lingula segment of the left upper lobe (1.8 cm × 0.8 cm) as well as the left wall of the bladder (Figure 4).

**Figure 5:**
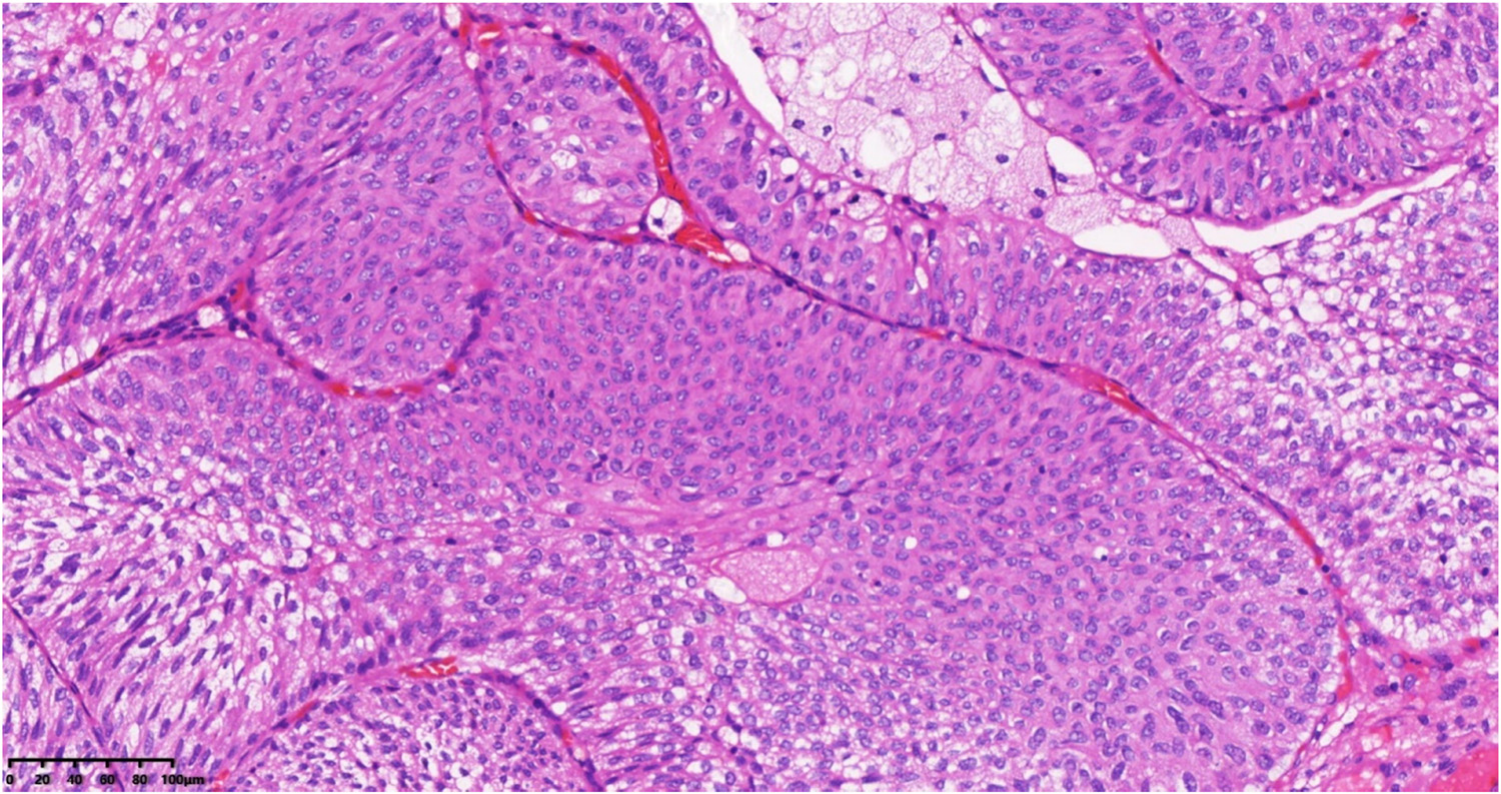
Photomicrograph (hematoxylin–eosin stain 200× magnification) revealed a low-grade no-ninvasive urothelial carcinoma of the bladder.

**Figure 6:**
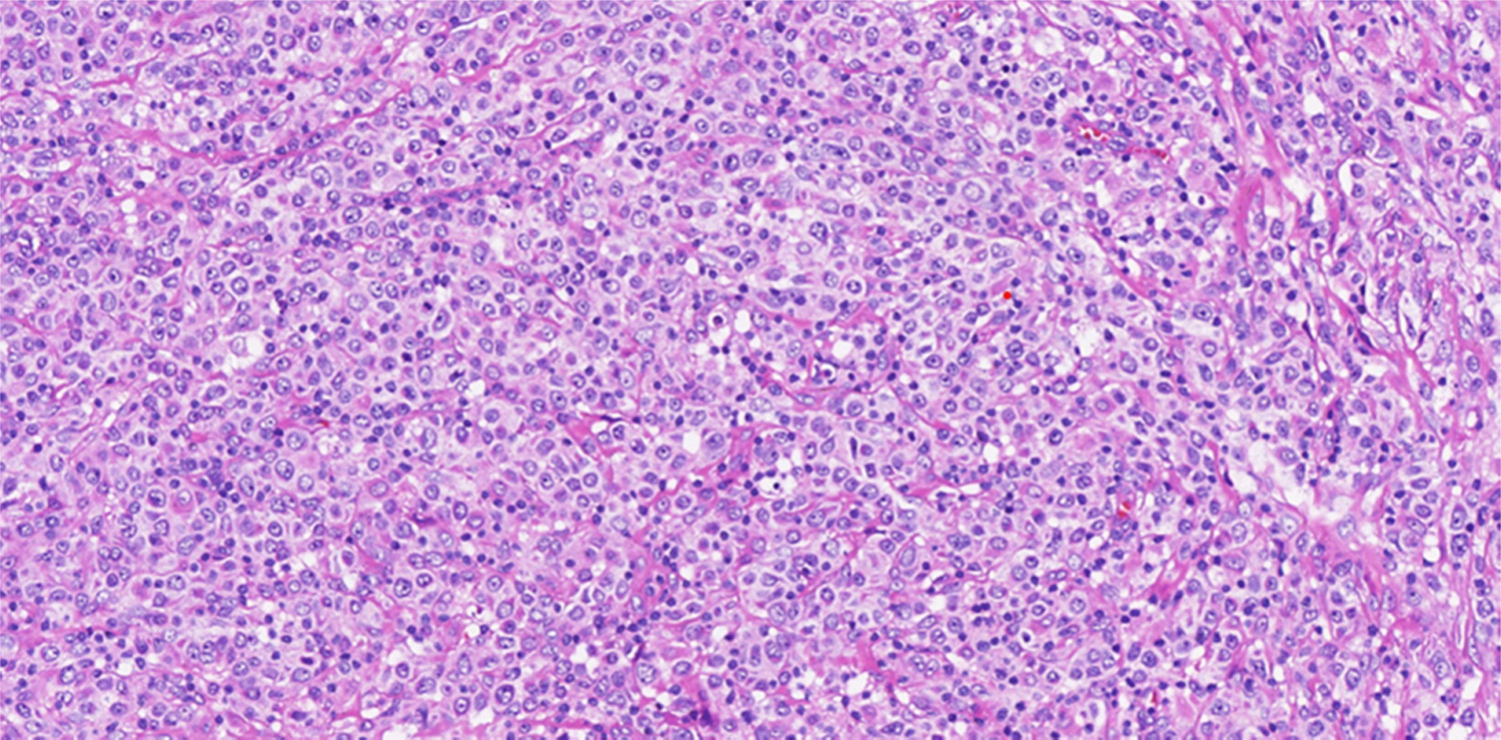
DLBCL, non-GCB subtype. Photomicrograph (hematoxylin–eosin stain 200× magnification) showed that the lymphoma cells were large, with relatively abundant cytoplasm and irregular cleaved nuclei.

**Figure 7:**
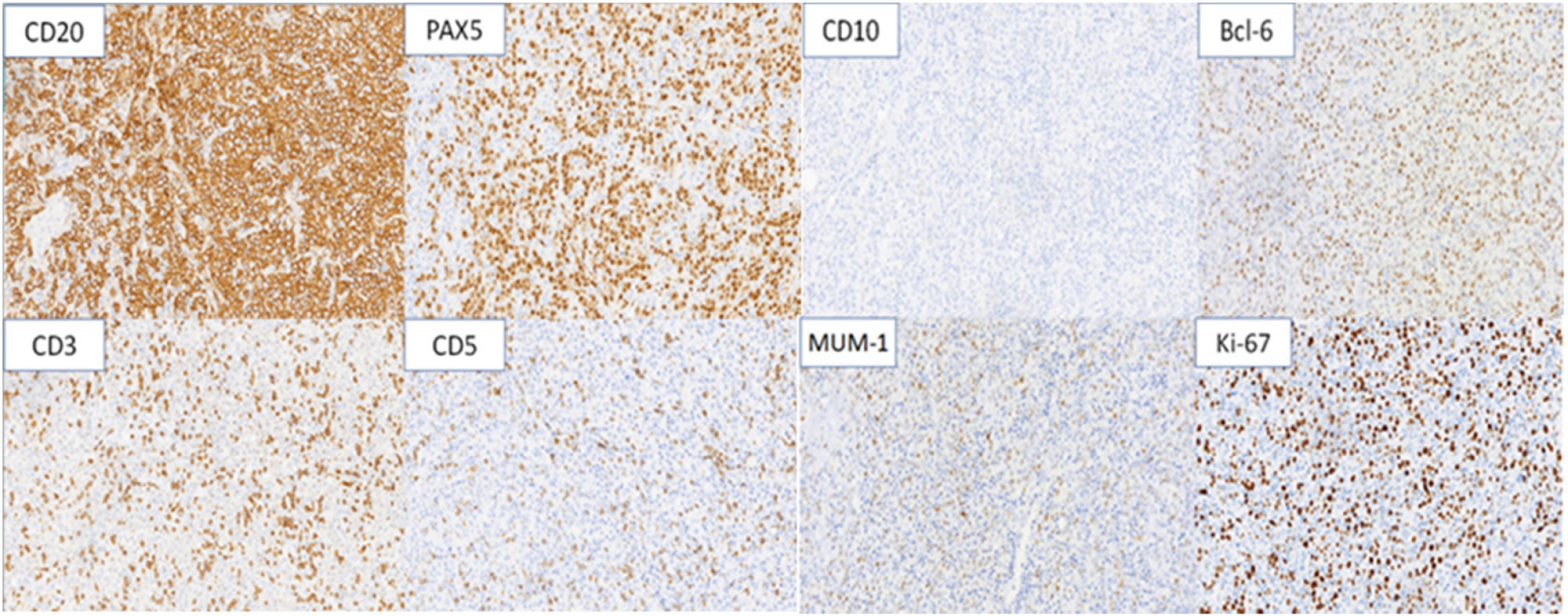
Immunohistochemical staining (200× magnification) of lymphoma cells was CD20 (+), PAX-5 (+), CD10 (–), Bcl-6 (+), MUM-1 (+), C-myc (10% +), Bcl-2 (30% +), MUM-1 (+), CD30 (–), CD3 (–), CD5 (–), EBER (–), and Ki-67 (60% +).

After deliberate discussion, to make the histological diagnosis of synchronous triple primary tumor, a cystoscopic biopsy was performed. Microscopic examination revealed a low-grade noninvasive urothelial carcinoma of the bladder (Figure 5). Laparoscopic lymph node biopsy showed DLBCL (Figures 6 and 7). Regarding the pulmonary nodule, it was difficult to obtain pathological results by CT-guided percutaneous nodule biopsy or tracheoscopy due to the location.

Considering the presence of these multiple malignant tumors, a multidisciplinary approach was needed. In a multidisciplinary conference, each malignancy was discussed in detail. The tumor board reached a consensus that noninvasive urothelial carcinoma of the bladder should be routinely treated with transurethral resection of bladder tumor (TURBT), whereas DLBCL should be treated with standard first-line R-CHOP immunochemotherapy regimens. For his excised bladder carcinoma, no further treatment after TURBT was needed. The pulmonary nodules that appeared on 18F-FDG PET/CT may be a third primary lung cancer or a secondary manifestation derived from systemic lymphoma. A watch-and-wait approach was managed considering that the nodule was small and the biopsy sample was difficult taken.

After one cycle of the R-CHOP immunochemotherapy regimen, the patient felt that the abdominal pain disappeared and that his appetite improved significantly. After 6 cycles of chemotherapy, 18F-FDG PET/CT showed that the lymphoma lesions were significantly reduced, and complete remission was achieved. However, at the same time, 18F-FDG PET/CT showed that the lesion in the lingual segment of the left upper lobe was larger than before (from 1.8 cm × 0.8 cm to 2.3 cm × 0.9 cm).

**Figure 8:**
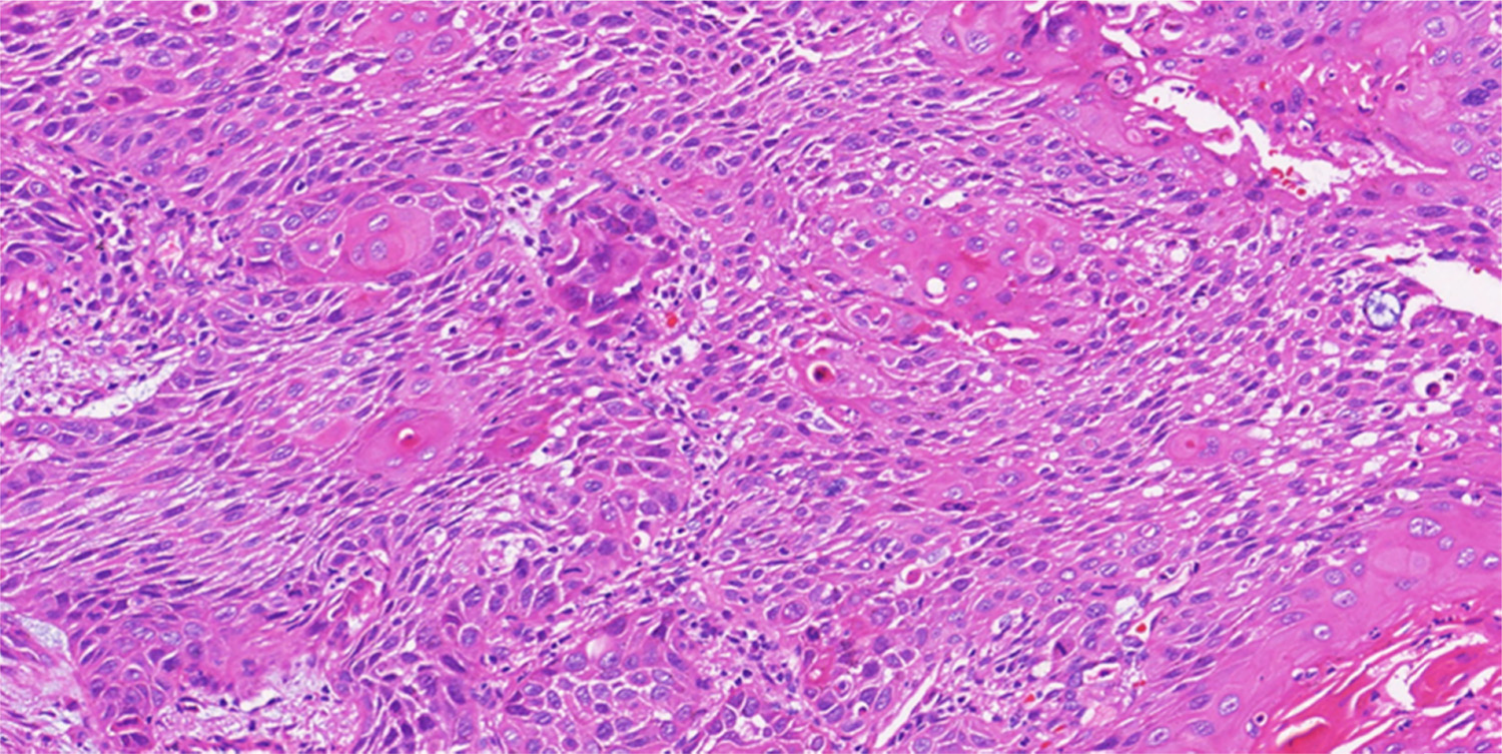
Pulmonary squamous cell carcinoma. Photomicrograph (hematoxylin–eosin stain 200× magnification) showed that well-moderately differentiated squamous cells.

**Figure 9:**
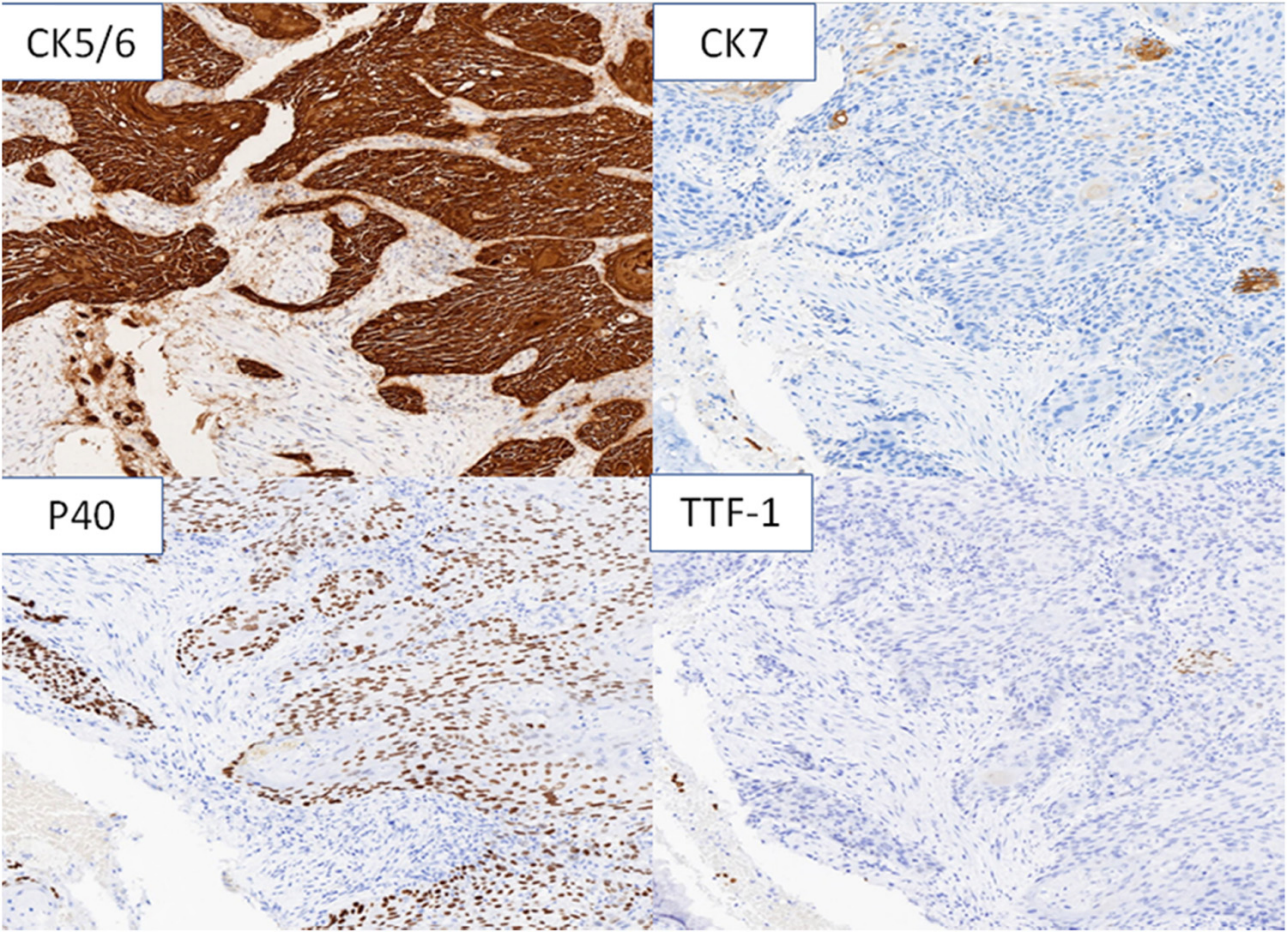
Immunohistochemical staining (100× magnification) of pulmonary carcinoma cells and immunocytochemistry was CK5/6 (+), CK7 (partially +), P40 (+), and TTF-1 (−).

**Figure 10:**
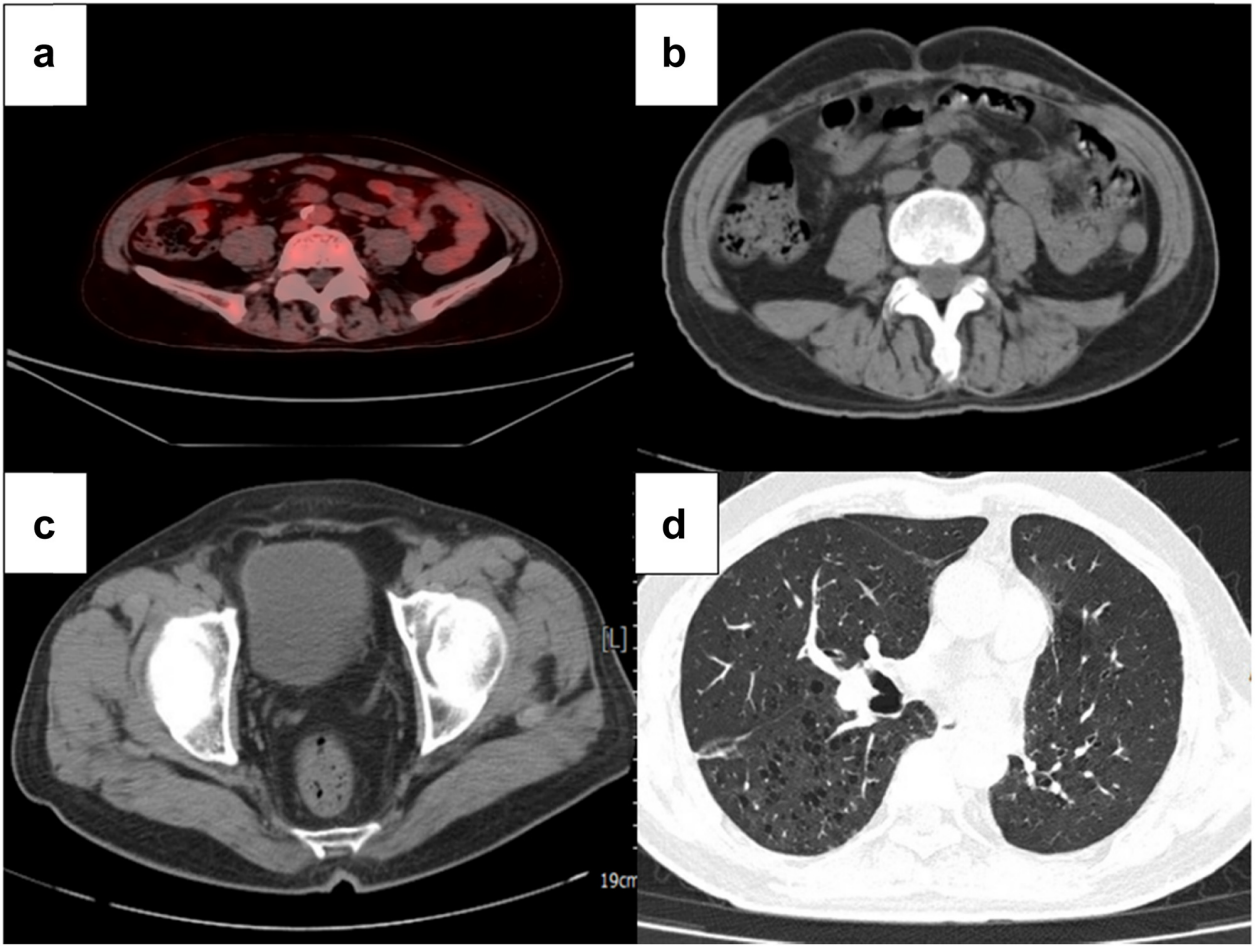
Follow-up CT scan and PET–CT scan showed no signs of disease relapse. (a) PET–CT scan performed at 1 month after standard chemotherapy, showing complete remission of lymphoma. (b) CT scan performed at 3 months after standard chemotherapy, showing complete remission of lymphoma. (c) CT revealed no signs of relapse in the primary site 3 months after TURBT. (d) CT revealed no signs of relapse in the primary site 3 months after surgical resection of the left upper lobe.


A further multidisciplinary assessment suggested that surgical treatment for the pulmonary lesion was the best treatment. Surgical specimens after left upper lobectomy showed well-moderately differentiated squamous cell carcinoma without pleural infiltration, vascular invasion, or necrosis. The tumor did not invade the surgical resection margins in the bronchial or vascular system or adjacent lymph nodes (Figure 8). Immunocytochemistry was CK5/6 (+), P40 (+), and TTF-1 (−) (Figure 9), whereas histopathological staging was pT1N0M0. Postoperatively, the patient was discharged after 18 days of rehabilitation. In addition, there were no plans for further adjuvant therapy. After pulmonary surgery, he was given two additional courses of R-CHOP chemotherapy. At present, a total of eight cycles of R-CHOP regimen have been completed. After 3 months of follow-up, there were no signs of disease relapse (Figure 10).

**Informed consent:** Informed consent has been obtained from all individuals included in this study.
**Ethical approval:** The research related to human use has been complied with all the relevant national regulations, institutional policies and in accordance with the tenets of the Helsinki Declaration, and has been approved by the authors’ institutional review board or equivalent committee.

## 3 Discussion

Synchronous triple primary tumors rarely occur. To date, multiple primary neoplasms have been reported in only a few cases [6]. Each malignancy must be histopathologically diagnosed and histopathologically distinct, and each tumor must be ruled out for the possibility of metastasis [7]. Cancers occurring within 6 months of the initial primary cancer are called synchronous cancers, while cancers occurring more than 6 months later are termed metachronous cancers [8]. It is still unclear what causes this association. Clinical negligence may lead to missed diagnoses, so a comprehensive and appropriate examination is critical for accurate diagnosis and treatment.

We report an extremely rare case of multiple primary tumors of bladder cancer, DLBCL, and lung cancer. In the present case, we first evaluated the probability of bladder cancer metastasizing to the abdominal lymph node or lung. However, pelvic lymph node metastasis in bladder cancer is frequent, followed by abdominal lymph nodes, and the 18F-FDG PET/CT scan demonstrated a round lesion with an irregular surface and pleural indentation in the upper lingual segment of the left lung, which were not metastatic signs. Therefore, we diagnosed the patient with synchronous primary lymphoma and lung cancer combined with bladder cancer. The results of the postoperative histopathology confirmed this hypothesis. Therefore, to plan a safe operation, it is important to examine the cancer carefully and stage it appropriately. The etiologies and epidemiologies of multiple primary tumors are under investigation, including common etiological factors, and the relationships between some tumors are well established [9,10]. Multiple primary tumors are predominantly seen in both the genitourinary and gastrointestinal tracts [11,12].

Multiple primary cancers can be caused by gene mutations and epigenetic modifications of chromatin [13,14]. The incidence of multiple malignant tumors in this 61-year-old male patient suggests that the cancer susceptibility gene may be mutated, accelerating the accumulation of somatic aberrations and promoting the development of cancer.

Overall, since simultaneous triple primary malignancies, including bladder cancer, lymphoma, and lung cancer, have not been reported, according to our findings, the association between such tumor types may not be one of them, and further research into the relationship between multiple primary tumor types, including common etiological factors, is warranted. There is no universal treatment protocol for multiple malignancies. A multidisciplinary approach is essential due to the wide variety of disease combinations and treatment options available.
